# Molecular Analysis of Echinostome Metacercariae from Their Second Intermediate Host Found in a Localised Geographic Region Reveals Genetic Heterogeneity and Possible Cryptic Speciation

**DOI:** 10.1371/journal.pntd.0002778

**Published:** 2014-04-03

**Authors:** Waraporn Noikong, Chalobol Wongsawad, Jong-Yil Chai, Supap Saenphet, Alan Trudgett

**Affiliations:** 1 Department of Biology, Faculty of Science, Chiang Mai University, Chiang Mai Province, Chiang Mai, Thailand; 2 Program of Applied Biology, Faculty of Science and Technology, Pibulsongkram Rajabhat University, Phitsanulok Province, Phitsanulok, Thailand; 3 Applied Technology in Biodiversity Research Unit, Institute of Science and Technology, Chiang Mai University, Chiang Mai Province, Chiang Mai, Thailand; 4 Department of Parasitology, Seoul National University College of Medicine, and Institute of Endemic Diseases, Seoul National University Medical Research Center, Seoul, Korea; 5 School of Biological Sciences, Medical Biology Centre, The Queen's University of Belfast, Belfast, Northern Ireland; George Washington University School of Medicine and Health Sciences, United States of America

## Abstract

Echinostome metacercariae are the infective stage for humans and animals. The identification of echinostomes has been based until recently on morphology but molecular techniques using sequences of ribosomal RNA and mitochondrial DNA have indicated major clades within the group. In this study we have used the ITS2 region of ribosomal RNA and the ND1 region of mitochondrial DNA to identify metacercariae from snails collected from eight well-separated sites from an area of 4000 km^2^ in Lamphun Province, Thailand. The derived sequences have been compared to those collected from elsewhere and have been deposited in the nucleotide databases. There were two aims of this study; firstly, to determine the species of echinostome present in an endemic area, and secondly, to assess the intra-specific genetic diversity, as this may be informative with regard to the potential for the development of anthelmintic resistance and with regard to the spread of infection by the definitive hosts. Our results indicate that the most prevalent species are most closely related to *E. revolutum, E. trivolvis, E. robustum, E. malayanum* and *Euparyphium albuferensis*. Some sites harbour several species and within a site there could be considerable intra-species genetic diversity. There is no significant geographical structuring within this area. Although the molecular techniques used in this study allowed the assignment of the samples to clades within defined species, however, within these groupings there were significant differences indicating that cryptic speciation may have occurred. The degree of genetic diversity present would suggest the use of targeted regimes designed to minimise the selection of anthelmintic resistance. The apparent lack of geographic structuring is consistent with the transmission of the parasites by the avian hosts.

## Author Summary

Infections by food-borne trematodes are estimated to infect over 40 million people worldwide, although infections by echinostomes make up only a portion of these cases, usually in regions where their prevalence is high. In South East Asia and in the far east of Asia, human infection is associated with cultural and dietary factors and the prevalence of infection may reach 50% in parts of Thailand, Cambodia, and Laos. Treatment is generally dependent on the use of praziquantel or benzimidazole drugs but with the occurrence of anthelmintic resistance to these compounds it would be desirable to have an understanding of the diversity present in the echinostome populations within a given locality. This study deals with the systematics of echinostomes and informs various aspects of the epidemiology of echinostomiasis which may aid the development of future control strategies.

## Introduction

Echinostomes are intestinal trematodes of humans and animals that are endemic to Southeast Asia and the Far East, i.e. mainland China, Taiwan, India, Korea, Malaysia, Philippines, Indonesia, and Thailand, and present a public health problem [1]. Human echinostomiasis has been attributed to at least twenty species belonging to eight genera (*Echinostoma*, *Echinochasmus*, *Acanthoparyphium*, *Artyfechinostomum*, *Episthmium*, *Himasthla*, *Hypoderaeumm*, and *Isthmiophora*) of digenea trematodes that use snails as intermediate hosts [2], [3]. Clinical symptoms of echinostomiasis include severe epigastric or abdominal pain accompanied by diarrhea, fatigue, anorexia, and malnutrition in humans [2], [3]. Numerous cases of human echinostomiasis have been reported in Japan (*E. cinetorchis*, *E. hortense*, and *E. japonicum*), India (*E. malayanum* and *Paryphostomum sufrartyfex*), and Thailand (*E. malayanum*, *E. revolutum*, *E. echinatum*, and *Hypoderaeum conoideum*) and are associated with the eating of raw fresh-water fish, snails, and tadpoles [4], [5], [6], [7]. In Thailand, stool examination is used to detect echinostome eggs in Thai women. The most common parasite found in both pregnant and non-pregnant women is *Opisthorchis viverrini*, (hookworm) while *Echinostoma* spp., *Strongyloides stercoralis, Taenia* spp., *Trichuris* and *Hymenolepis diminuta* are more rarely found under these circumstances [8].

The identification of the species of echinostomes has been based in the past on morphology with major clades being defined on the basis of the number and distribution of the collar spines [9]. However, due to a large number of morphological similarities, this has become difficult in many cases. Molecular techniques have revealed differences among morphologically similar parasites [10], [11], [12]. An additional benefit of these techniques is that they can permit the identification of species, strains, and populations from a small quantity of tissue from any stage in their life-history [10], [13]. Generally, an investigation of the phylogenetic relationships between echinostomes uses sequence data from the mitochondrial cytochrome c oxidase subunit 1 (CO1) and nicotinamide adenine dinucleotide dehydrogenase subunit 1 (ND1) genes [10], [12], [13], [14], [15]. These have been determined to be valuable for a more accurate estimate of echinostome diversity [13], [16]. The internal transcribed spacer region (ITS) of ribosomal RNA (rRNA) has also provided a means of discriminating between species that have similar morphology [17], [18]. In this study, molecular sequencing of the ITS 2 region and ND1 gene of echinostomes were utilized.

The treatment of echinostomiasis is largely reliant on two anthelmintics: albendazole and praziquantel. Both of these drugs have been associated with the development of anthelmintic resistance (AR) [19]. It is important that their application follows a regime which will minimize the development of anthelmintic resistance. The rate of development of AR is a function of the genetic diversity of the target echinostome population [20], consequently we were interested in determining the variety and genetic diversity of echinostomes in the area from which our patient population was drawn.

## Materials and Methods

### Sample collection

Echinostomes were obtained from naturally infected fresh water snail intermediate hosts; *Filopaludina martensi martensi*. They were collected from permanent and seasonal ponds from eight field sites in Lamphun Province in northern Thailand (Table 1). The metacercariae were removed from the snails by crushing and the parasites were examined for the presence/absence of the collar spines. Those metacercariae found to have collar spines were taken from each snail and were frozen immediately for later DNA extraction.

**Table 1:** Metacercariae collected from different localities.

Collection locality	Stage	Type of pond
Ban Hong	Metacercaria	Permanent
Ban Thi	Metacercaria	Seasonal
Lee	Metacercaria	Seasonal
Meaung	Metacercaria	Permanent
Mae Ta	Metacercaria	Permanent
Pa Sang	Metacercaria	Permanent
Toong Hua Chang	Metacercaria	Permanent
Weang Nong Long	Metacercaria	Seasonal

### Molecular studies – Extraction of DNA

DNA from all collected metacercariae was extracted as described in [21]. Briefly, 150 µl of 5% Chelex (Fluka) solution containing 10 µl of proteinase K (Sigma) at a concentration of 20 mg/ml was added to approximately 20 mg of trematode tissue. It was then heated at 55°C for 1 h, followed by gentle vortexing and heating at 95°C for 30 min, again followed by gentle vortexing. The mixture was centrifuged at 13,000 g for 10 sec. The supernatant was removed and stored at −20°C until it was to be used.

### PCR of the ITS2 region

Approximately 1000 base pairs (bp) of the ITS2 region were amplified by using the primers, forward BD1 (5′-GCT GTA ACA AGG TTT CCG TA-3′) and reverse BD2 (5′-TAT GCT TAA ATT CAG CGG GT-3′). The PCR conditions used were the same as those previously described in [10] with amplification steps as follows: 2 min initial denaturation at 94°C, followed by 39 cycles of 1 min DNA denaturation at 94°C, 1 min primer annealing at 57°C, and 1 min at 72°C for extension and a final extension of 72°C for 10 min.

### PCR of the mitochondrial ND1 gene

The amplification of ND1 and the PCR conditions used were those previously described in [10] with amplification steps as follows: 2 min initial denaturation at 94°C, followed by 39 cycles of 30 sec DNA denaturation at 94°C, 20 sec primer annealing at 48°C, and 1 min at 72°C for extension and final extension of 72°C for 10 min. Approximately 530 base pairs (bp) of the ND1 gene were amplified under these conditions by using the primers: forward JB11 (5′-AGA TTC GTA AGG GGC CTA ATA-3′) and reverse JB12 (5′-ACC ACT AAC TAA TTC ACT TTC-3′) as those described in [10].

Successful production of the amplicons and their quality was checked using agarose gel electrophoresis with ethidium bromide staining to visualize the ITS and ND1 products. All ITS and ND1 PCR products were purified using the Cleanup PCR Kit (Sigma) and were subjected to sequencing.

### Phylogenetic and network analyses

The raw sequencing data were assembled by Chromas Pro (Technelysium Pty. Ltd, Australia). Bio Edit software [22] was used to make sequence alignments which were compared to GenBank deposited sequences using BLASTN.

The sequence data produced in this study was combined with the data of 40 GenBank of echinostome sequences (ITS and ND1) and was aligned using BioEdit. Haplotype diversity and nucleotide diversity were both calculated by DNAsp [23]. Phylogenetic trees were generated for each gene using all sites with maximum likelihood. Branches were tested for all inferred trees using bootstrap analysis on 1,000 random trees. The relationship between the genetic diversity and the geographic distance within and among the species groups were calculated for each gene with MEGA version 5.0 [24]. The intra specific variation within each of the suggested clades and haplotype networks were constructed with statistical parsimony analysis for ND1 sequences (Network 4.6.1.1, fluxus-engineering.com, Fluxus Technology Ltd., UK, 2004). The 4× rule/K/θ ratio species criterion was applied to determine the likelihood of cryptic speciation [25].

## Results

**Figure 1:**
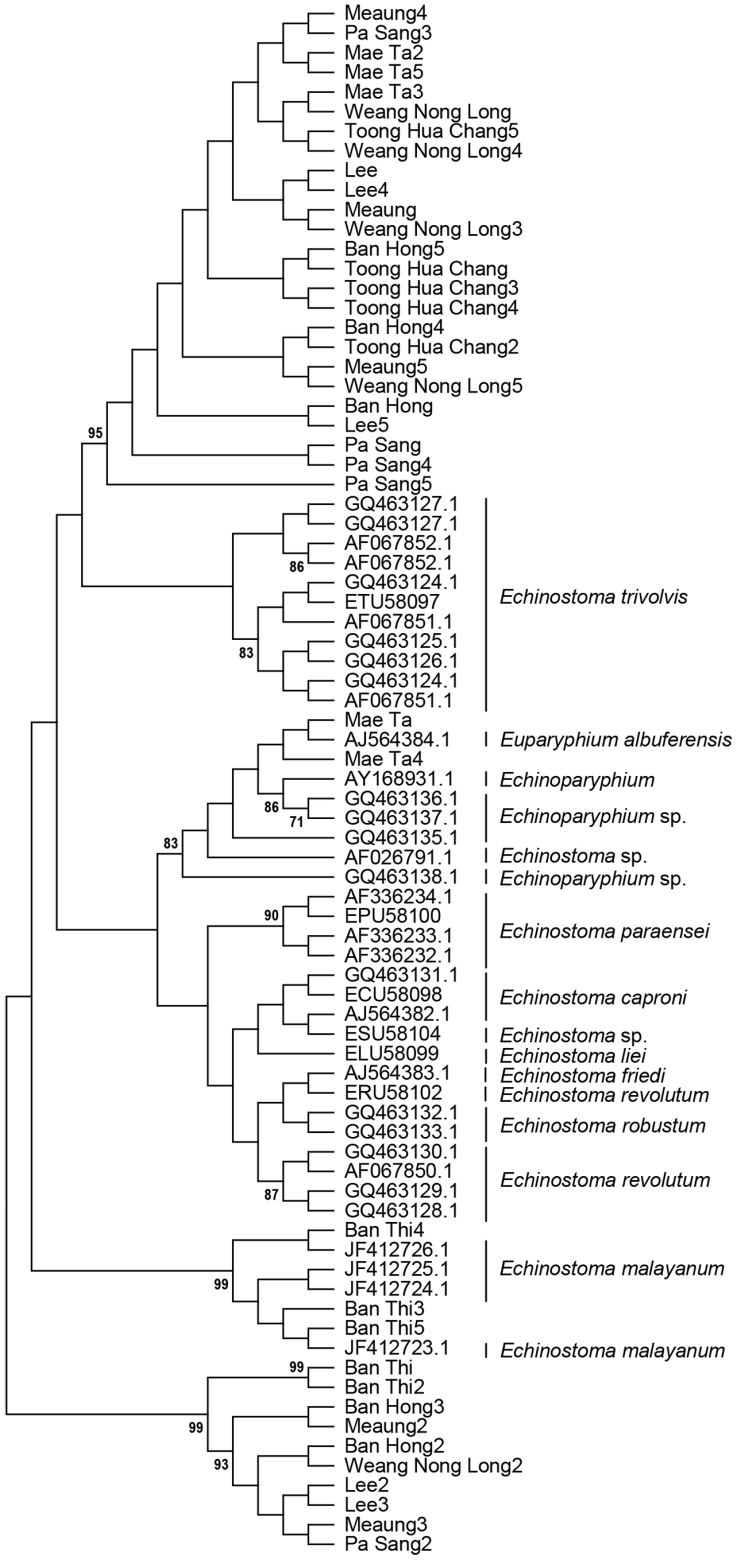
Maximum Likelihood bootstrap consensus tree with 1000 bootstrap iterations of ITS2. Only values higher than 70% are shown.

High quality sequence data (ITS2 and ND1) was obtained for forty metacercariae. Figure 1 shows the analysis of the ITS2 data obtained in this study, along with relevant sequences from GenBank as a Maximum Likelihood bootstrap consensus tree with 1000 bootstrap iterations. There is strong support (>70%) for monophyletic clades for *E. malayanum*, *E. revolutum*, *E. paraensei*, *E. trivolvis* and *Echinoparyphium* spp. Three of the samples from Ban Thi were grouped with the *E. malayanum* clade and two from Mae Ta with the *Echinoparyphium/Euparyphium* clade. The remainder of the samples formed two distinct monophyletic clades. The larger of these consisted of a single haplotype and both showed 98% identity within a range of Echinostoma ITS2 sequences. A Neighbor-Joining tree gave identical topology (not shown).

**Figure 2:**
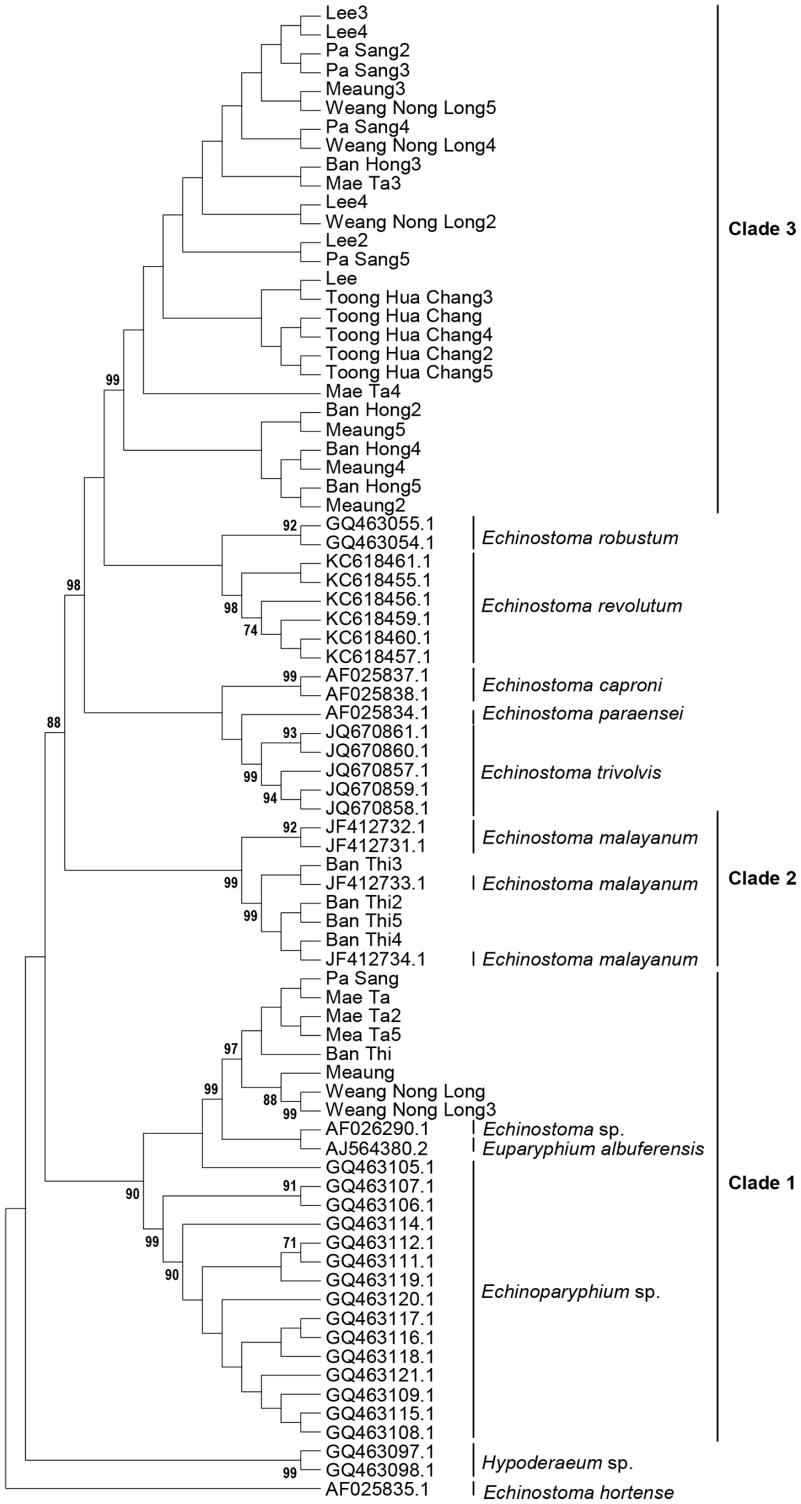
Maximum Likelihood bootstrap consensus tree with 1000 bootstrap iterations based on the ND1 sequences and relevant GenBank sequences. Only values higher than 70% are shown.

Figure 2 shows a Maximum Likelihood tree based on the ND1 sequences and relevant GenBank sequences. As with the ITS2 sequences, there was good support for monophyletic clades for *E. malayanum*, *E. revolutum*, *E. paraensei*, *E. trivolvis* and *Echinoparyphium* spp. In this analysis, four of the samples from Ban Thi were associated with *E. malayanum* and nine of the samples formed a monophyletic group with the *Echinoparyphium/Euparyphium* clade. The remaining twenty-seven samples (labelled “Clade 3”) formed a monophyletic group containing four haplotypes. The statistics associated with these samples are shown in Table 2.

**Table 2:** Genetic diversity of *Euparyphium albuferensis* and *Echinostoma robustum/trivolvis/revolutum-like*. *E. malayanum* were all the same haplotype – i.e. no genetic diversity.

Species	No. sequences	No. samples	No. Polymorphic sites	No. haplotypes	Haplotype diversity	Nucleotide diversity
*Euparyphium albuferensis- like*	9	8	11	3	0.556	0.00967
*Echinostoma –like*	27	27	3	4	0.655	0.00278
*Echinostoma malayanum*		5	0	1	Not applicable	Not applicable

In order to determine whether the echinostome-like samples were within the limits of the genetic diversity found in the *Echinoparyphium*, *E. trivolvis* and *E. revolutum* clades (there are insufficient sequences of *E. robustum* in the database to allow it to be included in this analysis), we applied the K>4θ test. The statistics associated with this calculation are shown in Table 3. This analysis indicated that the samples Ban Thi 2–5 should be considered as *E. malayanum*, but that the rest of the isolates, although sharing ancestry with either the *Echinoparyphium* or the *Echinostomatrivolvis/revolutum/robustum* clades, could be regarded as separate species by this criterion.

**Table 3:** Variables associated with the populations used to test compliance with the “4× rule” for speciation.

Population	Nucleotide diversity (π)	θ	θ×4	Sequence divergence between clades (K)	K≥4θ?
*Euparyphium/Echinoparyphium*-like	0.01190	0.01160	0.0464	0.21586	Yes
*E. malayanum*	0.15038	0.16268	0.65072	0.08904	No
*E. trivolvis*-like	0.00279	0.00195	0.0078	0.18251	Yes
*E. revolutum*-like	0.00279	0.00195	0.0078	0.15371	Yes

**Figure 3:**
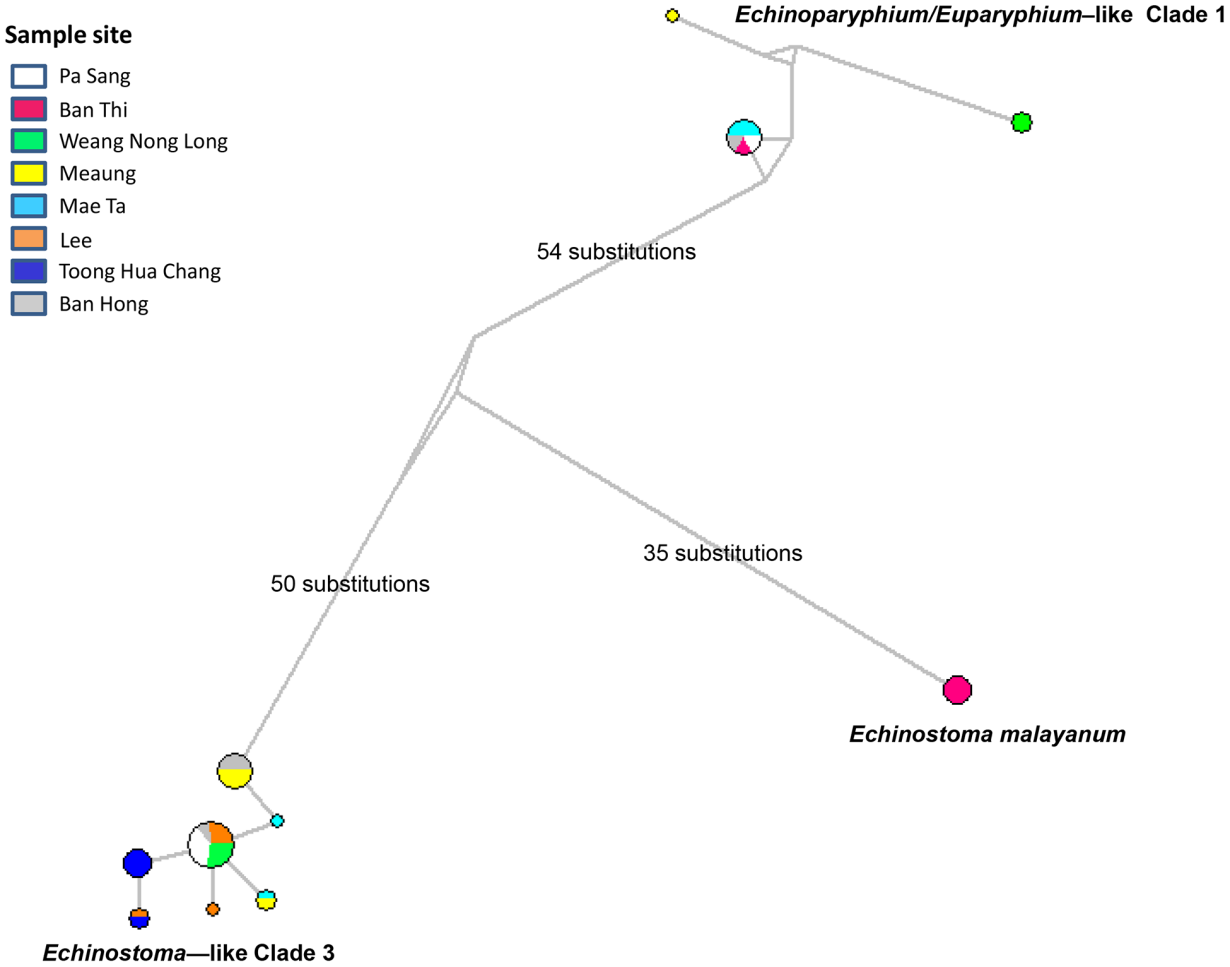
Median-Joining network echinostomes based on the ND1 sequences. Different colours represent the geographic origins of clades/groups identified in this study. The genetic distances between the clades and their geographic spread are shown.

There was considerable variability in the diversity of the species found at the different sites. Most sites had more than one species present and this parameter did not seem to be correlated with the permanence of the site. Figure 3 shows a schematic Median-Joining network constructed from the ND1 sequences. This analysis, using an alternative algorithm, confirmed the division of the samples into three clades. The genetic distances between the clades and their geographic spread are shown. The most frequent isolates were from the unidentified “echinostome-like” clade 3 grouping, which was found at six of the seven sites investigated.

## Discussion

The results presented in this paper provide the most extensive use to date of molecular techniques for the characterization of echinostomes from Thailand. Our results indicate that even small seasonal ponds may contain infected snails carrying a range of species. The samples in our study could be grouped into three distinct clades, *E. malayanum*, an *Euparyphium/Echinoparyphium*-like clade and an *Echinostoma trivolvis/revolutum*-like clade; worms identified as belonging to all of these groups have been shown to endemically infect humans in South East Asia [26]. Our findings are in agreement with those of [27], [28], who reported that *E. malayanum* and *E. revolutum* as being prevalent in Thailand. Although they recorded fixed genetic differences at 19% of the loci examined between Thai *E. revolutum* and those from the Lao PDR, they did not consider this as evidence of cryptic speciation as there was little divergence in the 200 bp of the mitochondrial cytochrome oxidase 1 (CO1) sequences for the worms from these two regions. In contrast, our analysis of the mitochondrial diversity of the “echinostome-like” clade 3 was based on approximately 800 bp of the ND1 region of the mitochondrial genome – this region is known to be more susceptible to changes [10], and thus may be more informative than the CO1 region. The analysis presented in Table 3 indicates that the “*Echinostoma*-like” clade 3 worms found in Thailand are genetically distant from *E. trivolvis* from North America and *E. revolutum* from northern Europe, and may be considered to constitute a cryptic species by the K>4θ criterion proposed by Birky [25]. Likewise the *Euparyphium/Echinoparyphium*-like Thai clade would appear to be a separate species from the North American *Echinoparyphium* spp., with which it was compared. As we were able to group some of our isolates with worms that were previously identified as *E. malayanum* from Thailand, this may indicate that on the continental scale there is geographical structuring of the Echinostomatidae family. It has been shown for other trematodes that the involvement of a highly motile host in the parasite's life cycle will reduce local geographic structuring [29]. All of the Echinostomatidae in this study are known to be capable of using avian species, such as ducks, as their definitive host, and Thailand is situated on the East Asian-Australasian Flyway, which has been implicated previously in the spread of zoonotic diseases [30]. Support for this suggestion may be given by the analysis of an *E. revolutum* isolate using the ITS 1 sequences [26], which indicated that it was more closely related to an Australian isolate than to those from North America.

In conclusion, we have shown that people living in a relatively small and homogeneous geographic area of South East Asia may be exposed to infection by at least three species of Echinostomatidae. There is sufficient genetic diversity present among these populations to allow for the selection of praziquantel resistance, as has occurred in the case of schistosomiasis [19], [31] and this finding emphasizes the need for targeted administration of chemotherapies.
